# The role of mentoring in academic career progression: a cross-sectional survey of the Academy of Medical Sciences mentoring scheme

**DOI:** 10.1177/0141076814530685

**Published:** 2014-08

**Authors:** Amy C Iversen, Nigel AJ Eady, Simon C Wessely

**Affiliations:** 1 Department of Psychological Medicine, Institute of Psychiatry, King’s College London, Weston Education Centre, London SE5 9RJ, UK; 2 Academy of Medical Sciences, 41 Portland Place, London W1B 1QH, UK

**Keywords:** mentoring, mentor, career, research, medicine, academic

## Abstract

**Objectives:**

To describe a successful mentoring scheme designed for mid-career clinician scientists and to examine factors associated with mentee report of positive career impact.

**Design:**

Mixed methods study including in-depth interviews and cross-sectional data collection via an online survey.

**Setting:**

Academy of Medical Sciences mentoring scheme set up in 2002 and evaluated in 2010.

**Participants:**

One hundred and forty-seven of 227 mentees took part in the study (response rate of 65%). Ten mentees, three mentors and eight stakeholders/scheme staff were selected to participate in in-depth interviews.

**Main outcome measures:**

Qualitative data: Interviews were transcribed, and free text was analysed to identify themes and subthemes in the narrative. Quantitative data: We examined the associations of reported positive career impact of mentoring by performing simple and multiple logistic regression analysis.

**Results:**

Mentoring success was determined by a variety of factors including reasons for selection (e.g. presence of a personal recommendation), mentee characteristics (e.g. younger age), experience and skills of the mentor (e.g. ‘mentor helped me to find my own solutions’) and the quality of the relationship (e.g. ‘my mentor and I set out clear expectations early on’).

**Conclusions:**

Our evaluation demonstrates that both mentor and mentee value mentoring and that careful planning of a scheme including preparation, training and ongoing support of both mentor and mentee addressing expectations, building rapport and logistics are likely to be helpful in ensuring success and benefit from the intervention.

## Introduction

The National Health Service (NHS) in the UK is a unique forum for the pursuit of medical research that advances understanding and leads to novel diagnostics and interventions for disease. A key requirement for the effective translation of research is a pool of talented bioscience professionals equipped with the necessary skills to exploit this potential.

This need for well-trained clinician scientists is well documented.^1–5^ Nonetheless, the future of academic medicine is often ‘on the critical list’.^6^ The development and implementation of an Integrated Academic Training Pathway by the National Institute for Health Research (NIHR) recognised this skills shortage by streamlining the funding schemes already available through major research funders such as the Medical Research Council, Wellcome Trust and others and provided young clinicians access to a clear roadmap into academic medicine.^7,8^ With this career pathway in place, it is now appropriate to consider what interventions may support young academics to progress.

Mentoring has been identified as a key mechanism to assist early career academics with career progression. In the US, formal mentoring is widely used and is part of organisational culture at all Academic Health Centers.^9^ Major funders such as the National Institute for Health consider the calibre and past performance of mentors to be as important as their protégés, and mentoring is formally enquired about in applications for training fellowships.^10^ In the UK, such formal endorsement of academic mentoring has been slower to embed; the major UK funders all request that junior academics are mentored, but there is no formal audit about whether this is occurring.^a^ This has meant that while there are pockets of good practice in some Higher Education Institutions,^11,12^ many aspiring early career academics have struggled to find suitable mentors to support them.

In 2002, the Academy of Medical Sciences (AMS) (Box 1) set up a Mentoring and Career Development Programme^b^ for early career academics^13^ to support what was seen to be a dwindling cadre of clinician scientists.^4^ The scheme is distinct in medical academia as its flagship component matches early and mid-career researchers, specifically academic Clinical Lecturers and Clinician Scientist Fellows, with independent mentors (Academy members) for one-to-one career development. A stakeholder describes the scheme as follows:

Access to some very successful people who can give a broader perspective, the bigger picture, because the trouble is that with day-to-day clinical supervision and day-to-day work in laboratories in research you can lose the big picture very easily. It is external and it’s from well respected people.

In this paper, we first describe mentoring as an intervention and go on to outline how the AMS scheme was set up. We then review the progress of the scheme to date and share the details of an evaluation recently undertaken, focusing on which factors are associated with reported positive career impact (mentee report).

**Box 1.** The Academy of Medical Sciences.

Founded in 1998, the Academy of Medical Sciences is the independent body in the UK representing the diversity of medical science. The Academy’s elected Fellows – over 1000 – are drawn from the fundamental biological sciences, clinical academic medicine, public and population health, health technology implementation, veterinary science, dentistry, medical and nursing care, and other professions allied to medical science as well as the essential underpinning disciplines including mathematics, chemistry, physics, engineering, ethics, social science and the law. It is the Fellowship, which provides the knowledge, influence and networks that enables the Academy to fulfil its vision of ‘improving health through research’. The Academy’s objectives are wide ranging, but a core objective underpinning its mission is ‘nurturing the next generation of medical researchers’.

## Mentoring as an intervention

Mentoring is defined as ‘off-line advice from one person to another to assist the recipient in making significant advances in their personal, professional or career development’.^14^ Modern mentoring models in Europe (often described as developmental mentoring) are distinct from traditional mentor–protégé relationships that have been historically widespread in academic medicine and based on models of patronage.^14^ In developmental mentoring, the emphasis is on mentees to find their own solutions to the challenges of career advancement rather than straight advice giving or ‘gifting’ of opportunities that is common in patronage. This form of support is more effective in the longer term because mentees are equipped with new problem-solving skills unlike patronage which offers ‘a hand up’ for the duration of the relationship but does not foster self-sufficiency.^15,16^ Mentoring is separate from supervision/appraisal which enables the mentee to speak more freely than they might with a research supervisor.

## The Academy of Medical Sciences mentoring scheme

The Academy’s one-to-one mentoring scheme was set up in 2002. At inception, the scheme was only open to Clinician Scientist Fellows.^c^ The scheme was devised by an expert^d^ with experience of both scholarship and practical delivery of mentoring schemes within medicine. Objectives of the scheme were to offer support and inspire potential clinical academics to develop independent research careers by providing access to objective guidance and mentoring from independent mentors apart from the mentee’s home institution, but also to enable Academy Fellows to keep in touch with the realities of being a early career clinical academic. All Academy Fellows are eligible to be selected as a mentor, but participation is voluntary. The Academy operates an ‘opt out’ system; unless a Fellow specifically requests not to mentor, they may be selected by potential mentees. Mentors are not required to attend any specific training before being chosen but are strongly encouraged to attend a half-day workshop covering key mentoring skills (approximately half take this up). Mentors are also signposted to online resources about mentoring. The scheme was deliberately designed to be ‘light touch’ and not to require ‘mandatory training’ because it was felt that this additional burden would deter senior mentors from taking part.

### Progress and development of the scheme

Regular informal reviews of the mentoring scheme were conducted from its inception. The first independent assessment of the mentoring scheme was carried out in 2006. The Department of Health and the National Institute for Health Research Trainees Coordinating Centre (NIHR TCC)^e^ reviewed the quality and relevance of the scheme and recommended that the scheme continue and be expanded. As a result of this endorsement and a resultant uplift in funding, the Academy was able to broaden the scheme’s remit and invited research-active Clinical Lecturers to select a mentor from the Academy’s Fellowship. Therefore, all UK Clinical Lecturers and Clinician Scientist Fellows are both now eligible for the scheme. Increased funding has also enabled a broader career development scheme to be implemented, which involves local workshops on career support/mentoring and opportunities for networking, all led by a dedicated staff.

Since 2002, over 350 mentor–mentee pairs have chosen to take part in the scheme. Sixty-nine percent of mentees involved in the scheme to date have been men, and 47% of the mentees have been based in London or the South East. At the time of writing, 166 of the now 1091 Academy Fellows have been selected as mentors and have agreed to participate. Eighty-four percent of the mentors on the scheme are men, reflecting the Academy’s membership (86% male) and the gender balance of the professoriate of the UK medical academic community.^f^ Eighty percent of mentors hold or have held a clinical role.

### Methods

In 2010, the Academy commissioned an independent evaluation of the scheme.^g^ The evaluation consisted of both quantitative and qualitative analysis. The main evaluation of the scheme was conducted via an online questionnaire sent to all mentees and mentors who had taken part in the scheme to date. To inform development of questionnaires and guide appropriate choice of measures, semi-structured interviews were conducted with scheme mentors (*n* = 1), mentees (*n* = 3) and Academy staff running of the scheme (*n* = 3). Interviews were also conducted with stakeholders^h^ (*n* = 5). The sample for each of the surveys was provided by the Academy from their records of those involved in the one-to-one mentoring scheme. All mentors and mentees were given the chance to opt out of the evaluation by the Academy before their details were passed to the evaluating team; one mentor opted out at this stage. The survey was open between 27 October 2010 and 13 December 2010, and three invitation reminders were sent out to mentors and mentees during this time period.

From this sample of participants, a small sample of mentees (*n* = 7) and mentors (*n* = 2) were selected for an in-depth telephone interview to provide further qualitative data. Interviews were transcribed, and free text was analysed to identify themes and subthemes in narratives.

### Analysis

The quantitative data are presented as descriptive data with numbers and percentages. Where we have used logistic regression to determine the association of one variable with another, we present the odds ratios and the 95% confidence intervals.

We used simple logistic regression to examine the associations between the dependent variable of interest – mentees reporting that the scheme has had positive impact on their career progression – and a variety of other variables identified *a priori*. For this analysis, we grouped those who reported a very or somewhat positive impact into a ‘positive’ group (*n* = 82), we disregarded those who had not indicated an impact either way (*n* = 9) and we compared the positive group with those who had reported no impact on their career (*n* = 48).

Then we grouped the independent variables of interest into six categories (Table 1) and produced a composite variable for each category. Each composite variable comprised a different set of individual variables for which simple logistic regression demonstrated a significant association with the dependent variable (career impact). For each composite variable for which a significant association with career impact was found, we have presented how the composite variable was derived.

**Table 1.** Categories derived from the data.

Category	Description
Selection	Factors that led (or may have led) the mentee to select the scheme or their chosen mentor
Mentee	Attributes of the mentee, including the duration of their involvement in the scheme, age etc.
Experience	The mentee’s evaluation of the experience of being mentored
Relationship	Relates to the relationship between the mentee and their mentor
Logistics	Factors such as the typical length of a session and who initiates contact
Support	Relates specifically to support that the Academy of Medical Sciences could provide for mentees

The five categories for which a significant association with career impact was demonstrated were then used in a multiple logistic regression analysis to see whether the associations that were observed when each of the individual composite variables was considered in isolation were preserved when the influence of other composite variables was taken into account.

## Results

### Characteristics of responders

One hundred and forty-seven of 227 mentees took part representing a 65% response rate. Most mentees held positions as either a Clinician Scientist Fellow (63%) or a Clinical Lecturer (30%). Fifty-six percent were based in London or surrounding areas, and 67% were men. Eighty-two of 166 mentors responded to the survey (50% response rate). Five mentors were deemed ineligible because they had not yet taken part in the scheme, giving an adjusted response rate of 51%. Of the 77 who remained, 91% were Professors and 29% were aged over 65 years. Eighty-one percent of mentors were men.

### Mentee report of impact

Nineteen percent of mentees reported that the scheme has had a very positive impact on their career progression to date, and among this group, satisfaction with the scheme was high. Forty percent said that it has had a fairly positive impact. The remainder reported that there had been no impact of mentoring (35%) or ‘did not know’ (6%). No mentees reported a negative impact.

Respondents reported that their mentors helped them by:

- inspiring greater confidence in their own abilities (59%);
- providing objective career advice and guidance (57%) to enable greater independence (60%); and
- fostering greater commitment to stay in academic medicine (53%).

Across six categories, we examined the associations of a wide variety of variables with the dependent variable (reported positive career impact of mentoring). The full range of variables explored is detailed in Table 1 of online Appendix 1. The significant associations are shown in Table 2. In brief, we found associations between all the categories we examined and report of positive career impact by the mentee. All of the categories remained significantly associated with the outcome variable when entered into a multiple logistic regression analysis (Table 3).

**Table 2.** Individual associations of mentee report variables with the dependent variable (positive impact on mentee’s career).

	No report of positive impact (*n* = 48)	Report of positive impact (*n* = 82)	Odds ratio (95% CI)
Selection			
I needed advice on a specific issue	2	15	5.15 (1.12–23.6)
I wanted general support on career options and decisions	27	65	2.97 (1.36–6.5)
I was impressed by the quality of the mentors available	7	33	3.94 (1.58–9.85)
S/he was recommended to me as being a good mentor	6	30	4.04 (1.54–10.61)
Two or more ‘selection’ statements	10	47	5.10 (2.24–11.6)
Mentee			
Aged 44 years or less at last birthday	35	78	7.24 (2.20–23.80)
Experience			
My mentor…			
served as a role model for me	25	72	4.53 (1.58–13.0)
encouraged me to talk openly about anxieties and fears	15	61	6.22 (2.71–14.3)
used his/her influence to my benefit	10	39	4.06 (1.67–9.83)
suggested specific strategies for achieving my career goals	30	79	14.0 (3.82–51.7)
challenged me to do things to develop new skills	13	59	7.66 (3.23–18.1)
shared personal experiences as an alternative perspective	18	61	4.71 (2.11–10.5)
demonstrated good listening skills	30	76	7.09 (2.35–21.4)
served as a sounding board for me	19	74	11.7 (4.33–31.5)
helped me to find my own solutions	24	76	23.8 (5.07–111)
Being mentored has helped me to …			
achieve a better balance between work and family life	1	18	16.4 (2.09–108)
achieve a better balance between research and clinical duties	3	38	14.6 (4.12–51.7)
develop networking skills	13	51	4.22 (1.88–9.50)
become more independent in my research career	11	61	15.5 (6.03–39.9)
stay in academic medicine	8	57	10.5 (4.17–26.5)
make a greater contribution to my research team	5	57	20.3 (6.87–59.8)
make a greater contribution to my hospital	2	29	12.1 (2.69–54.2)
achieve specific career changes and goals	4	68	74.4 (20.9–264)
have the confidence to apply for promotion	3	46	23.3 (6.48–84.0)
have more confidence in my own abilities	11	64	11.3 (4.60–27.5)
secure more research funding	1	34	34.0 (4.43–261)
publish more academic papers	3	30	9.74 (2.73–34.7)
develop more academic collaborations	9	57	10.6 (4.23–26.3)
Five or more ‘My mentor … ’ and seven or more ‘Being mentored … ’ statements	21	78	25.1 (7.90–79.6)
Relationship			
My mentor and I know and understand each other	11	60	5.43 (4.95–29.1)
My mentor is well-disposed towards me	28	80	17.1 (1.98–149)
My mentor and I set out clear expectations early on	13	60	7.53 (3.29–17.2)
The value I get is not sufficient given the time I put in	9	5	0.21 (0.07–0.69)
I was not able to spend enough time with my mentor	31	15	0.09 (0.04–0.22)
Three or more ‘relationship’ statements	17	74	16.9 (6.59–43.1)
Logistics			
My typical mentoring session lasts 1–2 h	14/41	41/78	2.40 (1.09–5.29)
Initial contact is shared between us	5/43	26/81	3.63 (1.28–10.3)
I have contact with my mentor at least once per year	25	77	14.2 (4.87–41.2)
Two or more ‘logistics’ statements	9	52	7.51 (3.20–17.6)

**Table 3.** Overall model.

Theme	No report of positive impact (*n* = 48)	Report of positive impact (*n* = 82)	Adjusted odds ratio (95% CI)
Selection	10	47	5.89 (1.77–19.7)
Mentee	35	78	9.35 (1.82–48.0)
Experience	21	78	7.30 (1.58–33.6)
Relationship	17	74	4.30 (1.12–16.5)
Logistics	9	52	6.21 (1.91–20.2)

## Mentor report of impact

There is also evidence that the scheme has had an impact on some of the mentors involved. Thirty percent strongly agreed that being a mentor has been worthwhile and 63% agreed somewhat. In particular, mentoring appeared to give some mentors a better understanding of the pressures faced by young academics, kept them up-to-date with the ‘bigger picture’ in their field and prompted them to think about their own career decisions and progress. Sixty-nine percent of mentors were willing to mentor again.

We examined the associations between mentor report of benefit from being a mentor (*n* = 53) and a variety of other variables that are detailed in Table 2 of online Appendix 1. The only significant association we discovered was that mentors who agreed with the statement ‘My mentee and I have/had come to know and understand each other well’ were more likely than those who disagreed to report benefit of participation in the scheme (odds ratio, 4.71; 95% CI, 1.11–20.1).

## Qualitative data

Mentees reported:

Having had the experience of successfully navigating through clinical medicine and science, my mentor has been instrumental in helping me structure my career when I hit a ‘road-block'.

When the challenges of combining clinical training and research clouded my judgment about future career steps, my mentor proved to be indispensable in making the most objective and adequate choice. Without his mentoring, I worry that I might have gone down the wrong path, rather than following my long-term aims. He was truly brilliant throughout.

So it was that kind of contact … with someone who basically took an interest in my career and in my development …we discussed lots of anecdotes, you know and about his experiences and again, that was also encouraging for someone who is starting out with the issues he’d faced and how he overcome them and how it was a complete nightmare when he was doing his clinical training, and again it was someone really inspiring who gave you a lot of energy and strength.

Those who had less successful mentoring relationships reported difficulties in a variety of different domains:

Partly my own fault, but I have not been proactive in asking to meet with the mentor specifically to discuss career progression. I often meet him briefly at academic meetings and in general he is very supportive but we haven't sat down together and talked about career progression.

We only met once, and a second meeting was cancelled at short notice. I didn't know enough at the time to realise I should have simply continued to make arrangements to meet, but after this didn't make contact again. The fault for this lies with me, not the mentor!

I liked and respected my mentor, but I don't feel that we were a good match. I think responsibility for this lies with me rather than them and I have recently taken steps to change my mentor.

My experience of the scheme was a negative one but I support the idea in principle and would very much like to have a mentor with whom I could meet on a regular basis and discuss career progression!

This was an expectation of the DoH Clinician Scientist fellowship scheme so I did it. I already knew the person, so the scheme per se changed nothing. I am not a fan of appointed mentors, either as a mentee or as a mentor. I think formalising a relationship ruins its mentoring capability.

I met him at a conference after e-mails had been exchanged and he made it clear that he was willing to be my mentor but that he had no real intention of engaging.

Mentors reported:

I think being a mentor has kept me more in touch with the realities and challenges for early career academics in navigating their way through a clinical academic career and highlighted the need to press for more resources to support career pathways in academic medicine.

It is a good thing to share your experiences with an ‘up and coming person' and having no ties to him/her (e.g. being responsible financially/educationally) gives a greater degree of freedom to really say what you think no codes, no partial concealment … just honest opinion!

It is enjoyable and provides a unique insight into the problems faced by my early career colleagues at a national level. I have got to know some very nice and interesting people! The process of mentoring someone else inevitably makes one reconsider your own position and reflect on what you want to do and achieve.

Mentees, stakeholders and mentors alike commented on the particular benefits of the Academy scheme:

It potentially offers access to a wider range of mentors than are available through institutional schemes. Also, I think it is very important to have a mentor from outside one's own institution to allow for impartiality and objectivity - the Academy's scheme offers this. (Mentee)

Collection of the finest academics in medicine … so as a clinical academic trainee, you couldn’t wish for a better set of individuals. I think particularly at the end of the Clinical Lecturer’s stage, you have a lot of uncertainty around what you want to do and how you should progress and you really value the opportunity to talk to experienced individuals who will give you good advice. (Mentee)

### Other themes identified from the qualitative and quantitative data

#### Selection and contact

The process of selecting a mentor was a clear area highlighted for potential improvement in the Academy scheme. Specifically, mentees requested more information about mentors and their interests and guidance in how to select a mentor. Both mentees and mentors agreed that it is the responsibility of mentees to make and maintain contact with their mentor. Contact between mentors and mentees, one to three times a year, was mainly face-to-face supported by email. Face-to-face meetings generally lasted 1–2 h and were mainly arranged as bespoke meetings.

#### Logistics of pairings

Both mentees and mentors emphasised the importance of the pair being based at different institutions in allowing the mentor to offer objective advice, although geographical distance was raised as a barrier to meeting by some mentees in the free text responses.

#### Training

Just over half of the mentors had received some training in mentoring either through the Academy or from elsewhere, and almost all mentors who had attended an Academy mentor development workshop found it very or fairly helpful. However, overall, mentors either did not see the need for training or did not regard training as important. Mentees, on the other hand, requested that mentors should have some training and would support training being offered to mentees as well.

#### Mentor’s role

Mentors saw their role as encouraging mentees to talk openly, offering an alternative perspective and acting as a sounding board, and they saw a clear distinction between the role as a mentor and that of a supervisor. Mentees reported that the mentor’s role was to suggest specific career strategies, help mentees to find their own solutions and to be good listeners.

#### Ending

Mentees were unsure when their mentoring relationship would end, and 28% of mentees would feel uncomfortable ending an unproductive relationship. Where mentoring relationships have ended, this tended to have been because the mentee had stopped contacting the mentor or had come to the end of a particular post.

## Discussion

### Key findings

The distinct nature of the Academy scheme, which allows early and mid-career academics to be paired with senior scientists outside their immediate institution and reporting line, has had clear benefits for the participants to date. Impact of mentoring appears to be determined by mentee’s age, the frequency and duration of meetings, mentee’s expectations of the mentoring relationship, the quality of the mentee–mentor interaction and the perceived role of the mentor, and all of these variables remained significantly associated with reported positive career impact when entered into a multiple logistic regression analysis.

### Limitations of data

The current data are limited in various ways. As the data are anonymous, it was not possible to pair mentor and mentee data. The response rate means that it is possible that response bias may have been introduced; it may be that those who did not respond had a different experience of the mentoring scheme from the participants in this study. We have collected data from mentors and mentees relatively early in their relationship; given that mentors and mentees only meet once or twice a year, it may be that we need to examine outcomes five years or more into the mentoring relationship in order to see the true benefit of the interventions. Finally, the data are cross-sectional, meaning that we cannot infer a direction of causality for the associations we report.

### Factors associated with success

Several key variables appear to be associated with a successful mentoring relationship. First, age seemed to matter with younger mentees reporting greater career impact than those aged over 45 years. This may be for a variety of reasons – it may be that the younger mentees joined the scheme more recently and the scheme has evolved over time, although this association persists when we controlled for ‘I am currently being mentored’ (data available from authors on request). It may instead be that those earlier in their careers are less likely to be critical of their senior mentors. Finally, it may be that younger scientists have more career flexibility and therefore benefit more from mentoring than those who are older and more ‘differentiated’ career-wise. Gender and career stage did not seem to matter; several commentators have suggested that women may preferentially benefit from mentorship,^17,18^ but these data do not support this.

Mentees who went into the mentoring relationship with a clear idea of what they hoped to gain, and who had received personal recommendations of a specific mentor, reported greater career impact – this is intuitive. In terms of mentor skills, serving as a sounding board and encouraging the mentee to think for themselves and find their own solutions appeared to be the most valued. The quality of the mentoring relationship emerged as an especially important association with reported career impact. Mentees who reported having adequate time with their mentors and who described a warm reciprocal relationship were more likely to report benefit. Meeting at least once a year and sessions that lasted at least an hour were also important; this information is useful for guiding new mentor–mentee pairs on our scheme. One interesting question that our study did not address relates to how formal mentoring schemes need to be in order to provide benefit. The majority of mentors on the scheme did not favour mandatory training, and instead this scheme has evolved as ‘light touch’. Future studies would profitably explore the impact of various features of a more formalised scheme such as training, contracts, ongoing supervision, etc. Currently, opinion is divided with some authors suggesting better outcomes from informal relationships,^19^ while others report no difference.^20^

## Conclusions

The AMS runs a successful mentoring scheme that pairs senior academics from across the UK with postdoctoral scientists from a wide variety of institutions. Feedback from both mentors and mentees is positive.

Academic medicine has always benefitted from a strong culture of informal ‘advice-giving’ from senior academics towards those seeking to develop their career. Mentoring harnesses this informal goodwill within a structured intervention to maximise the personal, professional and career development of early and mid-career academics. It is a low-cost, evidence-based intervention, which is easy to learn and as such has appeal in the current austere financial climate.

Our paper has highlighted that mentoring schemes are likely to be enhanced by careful planning and education of mentee and mentor. Training needs to allow for discussion about expectations of the mentoring relationship, as well as more practical logistics such as frequency of meetings.

## Declarations

### Guarantor

SCW

### Provenance

Not commissioned; peer-reviewed by David Metcalfe

## Notes

a The exception to this is the Wellcome Senior Investigator Scheme, which demands tangible evidence of high-quality mentoring.

b Formerly the ‘Mentoring and Outreach Scheme’.

c These new fellowships, designed for individuals with a higher degree such as a PhD or MD, were established based on the recommendation of an Academy working party chaired by Professor Sir John Savill, FRSE FMedSci. Published in 2000, the Savill report made a number of recommendations, intended to address perceived disincentives in following a clinical academic career, including the immediate establishment of the competitive clinician scientist track.

d Dr Jolyon Oxley.

e At the time called the National Coordinating Centre for Research Capacity Development (NCCRCD).

f As of February 2012, the Academy has appointed a taskforce to look at the representation of women in the Academy’s Fellowship. The group’s recommendations to the Academy’s Council in summer 2012, based on their study, are under discussion.

g The evaluating team was an independent social science research company.

h Representatives from NIHR, the Medical Research Council, the Wellcome Trust, Cancer Research UK and the Conference of Postgraduate Medical Deans of the UK.
